# Sheep scab transmission: a spatially explicit dynamic metapopulation model

**DOI:** 10.1186/s13567-021-00924-y

**Published:** 2021-04-12

**Authors:** Emily Nixon, Ellen Brooks-Pollock, Richard Wall

**Affiliations:** 1 School of Biological Sciences, University of Bristol, 24 Tyndall Avenue, Bristol, BS8 1TQ UK; 2 Bristol Veterinary School, University of Bristol, Langford House, Bristol, BS40 5DU UK; 3 NIHR Health Protection Research Unit in Behavioural Science and Evaluation at University of Bristol, Bristol, UK

**Keywords:** Control, Disease, Ectoparasite, *Psoroptes ovis*, Sheep movement, Transmission dynamics

## Abstract

Psoroptic mange (sheep scab), caused by the parasitic mite, *Psoroptes ovis*, is an important disease of sheep worldwide. It causes chronic animal welfare issues and economic losses. Eradication of scab has proved impossible in many sheep-rearing areas and recent reports of resistance to macrocyclic lactones, a key class of parasiticide, highlight the importance of improving approaches to scab management. To allow this, the current study aimed to develop a stochastic spatial metapopulation model for sheep scab transmission which can be adapted for use in any geographical region, exhibited here using data for Great Britain. The model uses agricultural survey and sheep movement data to geo-reference farms and capture realistic movement patterns. Reported data on sheep scab outbreaks from 1973 to 1991 were used for model fitting with Sequential Monte Carlo Approximate Bayesian Computation methods. The outbreak incidence predicted by the model was from the same statistical distribution as the reported outbreak data (${\chi }^{2}$ = 115.3, *p* = 1) and the spatial location of sheep scab outbreaks predicted was positively correlated with the observed outbreak data by county ($\tau$ = 0.55, *p* < 0.001), confirming that the model developed is able to accurately capture the number of farms infected in a year, the seasonality of scab incidence and the spatial patterns seen in the data. This model gives insight into the transmission dynamics of sheep scab and will allow the exploration of more effective control strategies.

**Supplementary Information:**

The online version contains supplementary material available at 10.1186/s13567-021-00924-y.

## Introduction

Psoroptic mange (sheep scab) is an important livestock disease caused by a hypersensitivity response in sheep to the faecal material of the parasitic mite *Psoroptes ovis* [1]. It causes chronic animal welfare issues associated with persistent pruritis and excoriation, resulting in wool loss and skin damage, and may lead ultimately to the death of the infested animal unless treated. This disease affects sheep farming systems worldwide, although not Australasia where it was successfully eradicated [2].

Scab was eradicated in Great Britain in the 1950s but was inadvertently reintroduced in 1972. Between 1972 and 1976, regional control methods were used to try to control outbreaks of scab, but these were unsuccessful, and the disease became endemic [3]. National control measures, based on the prophylactic use of organophosphate, and also initially pyrethroid dips, were then introduced to manage the disease. These succeeded in keeping farm outbreaks of scab below 100 per year [3] but were unsuccessful in re-achieving eradication. The failure to re-eradicate the disease, associated with concerns over human and environmental exposure to acaricides, led to deregulation in 1992 when compulsory national prophylactic treatment was abandoned. Once national statutory measures were lifted, the prevalence of scab increased exponentially before reaching a plateau at an estimated 7–8 thousand farm outbreaks per year [4]. Sheep scab is currently estimated to cause economic losses to farmers in Great Britain of about £GBP 78–202 million per year [5].

The development of new, effective interventions for scab may be particularly important in the coming years given recent reports of resistance in *P. ovis* to one of the main classes of parasiticide used to treat the condition, the macrocyclic lactones [6, 7]. Although no resistance has been reported yet to organophosphates, this is the only other licensed treatment available in Great Britain and it is important that more targeted scab management strategies help to preserve its efficacy.

Epidemiological modelling has been used successfully to understand the transmission of a range of sheep diseases: scrapie [8], foot and mouth [9, 10], bovine spongiform encephalopathy [11, 12] and bluetongue [13]. However, while a great deal is known about risk-factors [14], there have previously been no comprehensive epidemiological models of sheep scab to help guide intervention. Although there have been many attempts to improve the control of scab by the government [3] and industry [15] at a local, regional and national level in Great Britain, none of these have been effective and it also remains an endemic disease in many other European, African, South American and Asian countries despite multiple attempts at control [2]. Modelling could give insight into the reasons for the failure of past interventions and identify optimum strategies to be used in the future.

The successful development and parameterisation of models relies on good quality data [16]. However, where limited data are available or uncertainty exists for certain parameters, Approximate Bayesian Computation (ABC) can be used with the information that is known (priors) to estimate posterior distributions for these parameters [17]. An efficient ABC method, sequential Monte Carlo (SMC ABC), first developed by [18], improves upon other Markov chain Monte Carlo methods by approximating the posterior in a progressive manner. This approach has been improved further by others [19–23] with the algorithm proposed by [22] shown to be the most efficient when applied to a toy example and a complex social model.

The aim of this study, therefore, was to develop a stochastic spatial metapopulation model which incorporates both within-farm and between-farm transmission of scab and can be adapted for use in any geographical region, exhibited here using data for Great Britain and fitted using SMC ABC; the objective was to show that the model developed could be capable of helping shape future interventions of sheep scab by simulation of potential control strategies.

## Materials and methods

### Model description

**Figure 1:**
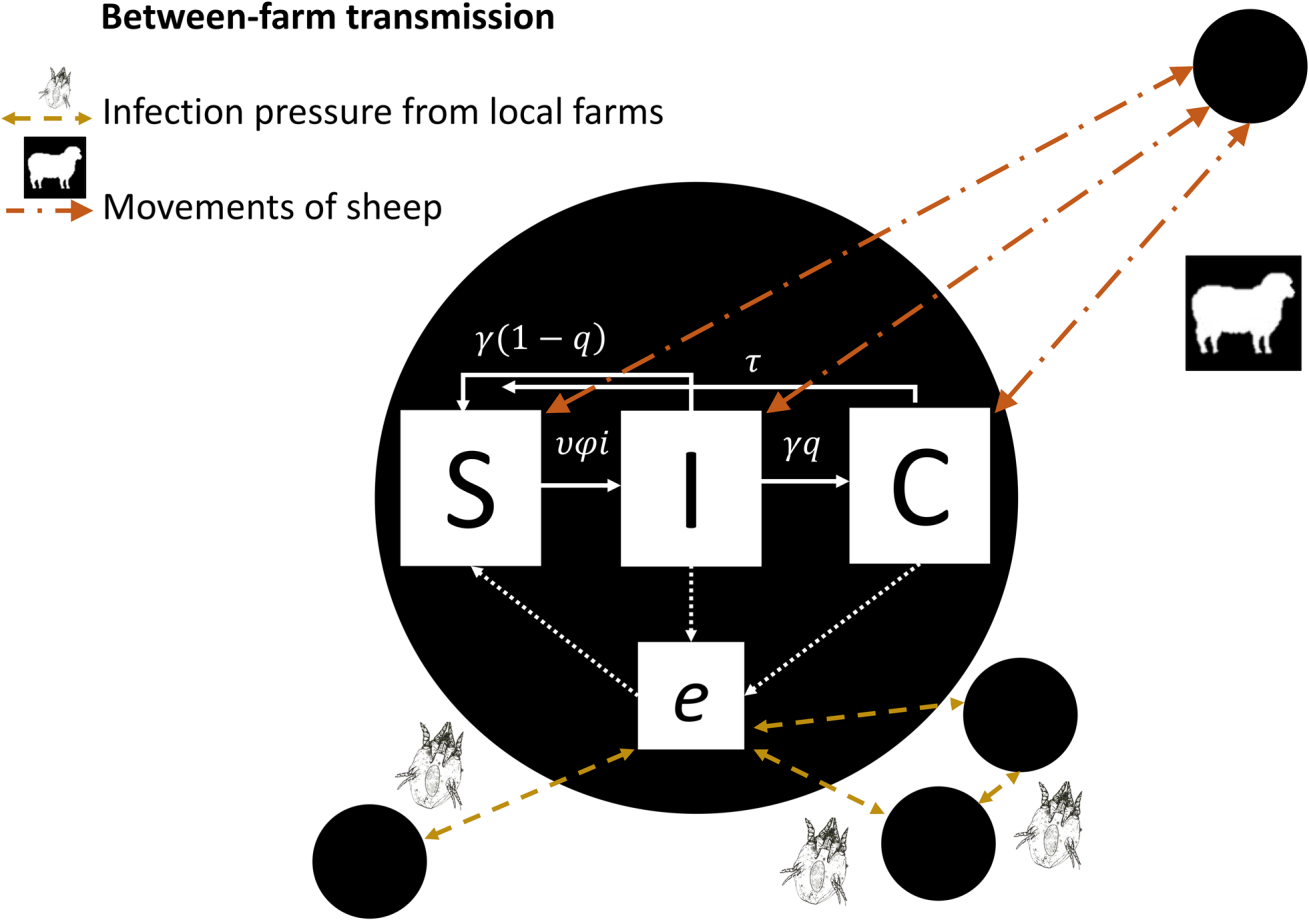
**Schematic of stochastic metapopulation of transmission of sheep scab within and between farms.** Within a farm (black circles), there are sheep which are susceptible (S), infected (I) or carriers (C) that can move between these states in the directions indicated by the block white arrows and at the rates defined in Table 1. There is an environmental compartment (*e*) within each farm which has an infectious pressure determined by shedding of *P. ovis* mites by infected or carrier sheep within the same flock or from infected and carrier sheep from contiguous (geographically close) flocks (represented by the white and yellow dashed arrows, respectively). A farm was considered to be geographically close if it was within a 2 km radius (Table 1). Susceptible sheep become infected via transmission from the environmental compartment. Equation (1) describes the rate of change of the environmental infectious pressure over time. Sheep scab transmission between farms that are not contiguous can occur via long distance movements of sheep (orange dotted and dashed arrows) where sheep in the movement batch are infected or carriers.

The model (Figure 1) was written by adapting and writing C and R code in the existing R package SimInf [24, 25]. The model code is available at [26] and the changes made to the SimInf package are described in Additional file 1.

The true locations of holdings and data on sheep movements in 2010 in Great Britain were used to create a contact network of farms and the model was fitted over time using SMC-ABC methods. Within-farm transmission was modelled using compartments for Susceptible (S), Infected (I) and Carrier (C) sheep (Figure 1), where susceptible animals are uninfected, infected animals show clinical signs and carriers are asymptomatic infectious animals. An additional environmental compartment (*e*) was added which could be contaminated with *P. ovis* mites*.* Susceptible animals become infected via transmission from the environmental compartment, which has an infectious pressure determined by *P. ovis* shedding from infected or carrier sheep within the same flock or from infected and carrier sheep from a geographically contiguous flock (Figure 1). A time-dependent environmental infectious pressure $\varphi i(t)$ is used to model the environmental compartment within each holding *i* at time *t* and is assumed to be equally spread across the entire area of the holding. The area of the holding is assumed to be proportional to the number of individuals within the holding. The rate of change of the environmental infectious pressure $\varphi i(t)$ over time is described as:

1 $$\frac{d{\varphi }_{i}}{dt}=\frac{\alpha {I}_{i}\left(t\right)+\varepsilon \alpha {C}_{i}(t)}{{N}_{i}(t)}+\sum_{k}\frac{{\varphi }_{k}\left(t\right){N}_{k}\left(t\right)-{\varphi }_{i}(t){N}_{i}(t)}{{N}_{i}(t)}*\frac{D}{{d}_{ik}}- \beta \left(t\right){\varphi }_{i}\left(t\right),$$

where *i* is a sheep holding and *k* is a sheep holding that is contiguous to *i* such that transmission of scab between the holdings by direct contact is possible. Equation (1) is adapted from Equation (2) in [24]. The first term describes the contribution of infected and carrier sheep to the environmental infectious pressure within a holding and has been adapted to include carriers of disease with the addition of $\varepsilon {\alpha C}_{i}\left(t\right)$ which includes a scaling ($\varepsilon$) for the transmission rate for carriers on the shedding rate for infectious individuals (α), since experimental data has suggested that carriers harbour fewer mites than acutely infected individuals [27]. The second term describes the contribution of environmental infectious pressure from carriers and infected individuals in all holdings contiguous to holding *i*. This was assumed to decrease as the magnitude of the Euclidean distance between holding *i* and a holding *k* (*d*_*ik*_) increases. The third term describes the decay of the environmental infectious pressure, as mites off host eventually die. All parameters are defined in Table 1. The direction and nature of the transitions between the compartments within a node *i* are given in Figure 1.

**Table 1:** **Parameters used in a metapopulation model of sheep scab (see Additional file ****1**** for sources).**

Parameter	Description	Value used in model
$\alpha$	The daily rate of *P. ovis* shedding per infected individual that contributed to environmental infectious pressure	Prior (0,0.012, uniform)
$\varepsilon$	The scaling rate for the contribution of carriers to environmental infectious pressure	$\frac{1}{3}$
*D*	Spatial coupling between holding *i* and neighbour *k*	0.5642857
${d}_{ik}$	The Euclidean distance between holding *i* and neighbour *k*	All distances up to 2 km radius
${S}_{i},{I}_{i, }{C}_{i}, {T}_{i}$	The number of infected sheep at holding *i*	**–**
$${N}_{i}$$	The total number of sheep in all compartments ($${S}_{i}+{I}_{i}+{C}_{i})$$	**–**
$\beta$	The decay rate of the environmental infectious pressure	Prior (0.02, 0.08, uniform)
$\upsilon$	The indirect transmission rate from the environmental compartment to susceptible sheep	Prior (0,0.0006, uniform)
$\gamma$	Recovery rate for infected sheep	$\frac{1}{77}$ days^−1^
$q$	The proportion of acute infections that become carriers ($q$)	$\frac{1}{2}$
$\tau$	Recovery rate for carriers	$\frac{1}{653}$ days^−1^
$\varphi i(t)$	A time-dependent environmental infectious pressure at holding *i*	**–**

All transitions are modelled as continuous-time discrete Markov chains (CTMC) using the Direct Method [28], as described fully in [29] and [25]. All simulations presented here started on the 1^st^ January and were run for a simulated theoretical year. Natural births and deaths are not included in any simulations, due to the comparatively rapid timescales associated with infection and transmission. Movements of sheep between farms are specified as external transmission “events” in SimInf. The dates for each movement were converted into timesteps, where each day represents one timestep. The movement events are executed when the simulation in continuous time reaches the timestep where movements were specified. Individuals that are to be moved between nodes are sampled randomly from the source node across all disease compartments in that node and allocated to the corresponding disease compartment in the destination node. A full description of how events (births, deaths, movements and treatment) are implemented in SimInf is given in [25].

### Scab incidence data

Sheep scab in Great Britain was notifiable between 1973 and 1992, after which there was no consistent national collection of scab outbreak data. The model was therefore fitted to data from the period between reintroduction and its deregulation (1973–1992). Data on the locations and dates of sheep scab outbreaks were taken from two sources. Data for 1973–1982 were obtained from historic outbreak records as published by [3]. These were derived from data held by the Ministry of Agriculture, Fisheries and Food (MAFF) Veterinary Laboratories Agency (VLA), Addlestone, UK and included the location of the farm infected and the date at which *P. ovis* infestation was confirmed by skin and wool scraping diagnosis at the MAFF VLA. Secondly, data for 1983–1992 were obtained data collected by the MAFF State Veterinary Service [3]. The latter data included the Ordinance Survey (OS) Grid references of infected sites and the date at which infestation was confirmed by skin and wool scraping diagnosis at the MAFF VLA. Changes in scab treatment policy were not explicitly included in this version of the model, but since the model is fitted to national outbreak data where scab treatment at a regional or national level was compulsory, treatment is implicit within the model outcomes.

### Sheep movement data

Movement data from 1973 to 1992 period were not available for Great Britain but, given that the movement of sheep from upland to lowland follows a generally consistent pattern from year to year as animals are sold, overwintered or taken for slaughter [30], detailed sheep movement data could be obtained for 2010 and were used here to provide a representative pattern. These data were provided by the Animal and Plant Health Agency (APHA) and were a combination of sheep movements recorded in the Animal Movement Licensing System (AMLS), maintained and administered by the Department for the Environment, Food and Rural Affairs (DEFRA) and the Scottish Animal Movement System (SAMS) which is run by the Scottish Executive Environment and Rural Affairs Department (SEERAD). In 2010, sheep movements in England, Wales and Scotland were recorded in accordance with the Sheep and Goats (Records, Identification and Movement) Order 2009. The data included the county parish holding number and coordinates of the departure and destination locations, as well as the number of sheep moved. The data show a strong seasonality in the number of batches of sheep moved and in the total number moved, with the number of batches of sheep moved remaining fairly stable between week 1 to week 30 (where week 1 is the first week in January 2010) at a mean of 12 864 batches moved per week (range = 8760–16 867, median = 13 998, IQR = 13 525–14 611). There was a general upward trend in the number of movements per week from week 30 and peaking at week 37 (early September) with 37 053 movements and then a gradual decline in the number of movements per week throughout the rest of the year. This pattern was closely followed in the total number of sheep moved, with 1.3 million sheep moved in the peak week (week 37).

### Holding data

Agricultural survey data provided by the Animal and Plant Health Agency (APHA) for England and Wales (June Census of Agriculture and Horticulture, 2010) and Scotland (June Agricultural Census, 2010) were used along with the sheep movement data to calculate the initial number of sheep at each holding at the start of the model simulation. These data also included the location of each holding. Markets and other holdings that were not included in the agricultural survey data, but were included in the movements data, were added to the holding data and were assumed to have no sheep at the start of the simulation, since markets tend to not have sheep residing there for long periods of time. As the agricultural survey takes place in June, but the movements start in January, adjustments to the numbers of sheep at each holding were made so that the agricultural survey data was reconciled with the sheep movement data. A full description of this process can be found in Additional file 1.

### Model parameterisation

The locations of sheep holdings, as eastings and northings, were used to determine which farms were considered to be neighbours in the model using the distance_matrix() function in SimInf. This function calculated *d*_*ik*_ for each farm *i* and each of the contiguous neighbours *k.* It was assumed that farms within 2 km of each other (in the distance matrix function, cutoff = 2000) were geographically close enough to be considered contiguous and for direct transmission to occur via the environment (Table 1).

Six model parameters were calibrated from the literature (Table 1). The remaining three parameters for which limited published data was available were estimated using Sequential Monte Carlo Approximate Bayesian Computation (SMC-ABC). These were the daily contribution to environmental pressure per infected individual (α), the decay rate of the environmental infectious pressure (β) and the indirect transmission rate from the environmental compartment *(e)* to susceptible sheep in holding i (υ). The prior distributions for the parameters were calculated as described in Additional file 1 and are given in Table 1. The ABC_sequential() function from the R package “EasyABC” [31] was used with the Lenormand method [22] where 50% of particles were kept at each step of the algorithm. The stopping criterion (predetermined threshold for accepted particles-Paccmin) was 0.11, as this value was found to have good convergence in preliminary exploration.

The model was fitted to the historical data on scab notifications from 1973 to 1991. The targeted summary statistics were the number of farm outbreaks for each week summed across all years from 1973 to 1991 (median = 19, mean = 26.9, IQR = 10–38.3, range = 3–76) and the mean yearly incidence (73.7 outbreaks) as reported to the Ministry of Agriculture, Fisheries and Food (MAFF) (median = 67, IQR = 43–97.5, range = 18–174). These summary statistics were chosen because the number of farm outbreaks in any given week was low and highly variable from year to year. The model summary statistics used were the weekly incidence of scab outbreaks across all holdings in the model in a one-year period multiplied by 19 (the number of years in the targeted weekly data) and the yearly incidence. The weekly incidence data were divided by 19 before being presented.

### Model simulations

The simulations started on the 1^st^ January and were run for a simulated theoretical year. At the start of the simulation, the number of susceptible sheep in each holding was set to be equal to the number of sheep reported in the holding data. The number of sheep in the other compartments in each holding were set to 0, other than in holdings which were assumed to be initially infected in the simulation. The initially infected farms were selected based on the farms that were reported to be infected in December 1975 according to the MAFF data (*n* = 33, after processing, 6846 sheep infected in total across all farms). It was assumed that all the farms reporting infection in December 1975 were still infected on the 1^st^ January (the simulation start date). Data on infected farms in December 1975 were selected to initialise the model because this was almost three years after scab was re-introduced into Great Britain, but before any national control programs had been implemented. Therefore, initialising the model with these farms gives a good indication of the number and location of farms that were infected once scab became endemic and before any national control methods had been used.

### Statistical analysis

A Pearson’s Chi-squared test for count data was carried on the mean weekly incidence using the “chisq.test” function from the “stats” (v3.6.1) package in R [32] in order to test the null hypothesis that the model simulation results and the reported MAFF data were from the same distribution. This was assumed to be the case where the *p* value was greater than 0.01. The *p* value was calculated using a Monte-Carlo test procedure with 2000 replicates as described by Hope [33]. Kendall rank-order correlation test was used to test the association between the percentages of the number of years (for the MAFF data) or simulations (for the model results) where outbreaks occurred in each county, using the cor.test() function from the “stats” (v3.6.1) package in R [32].

## Results

**Figure 2:**
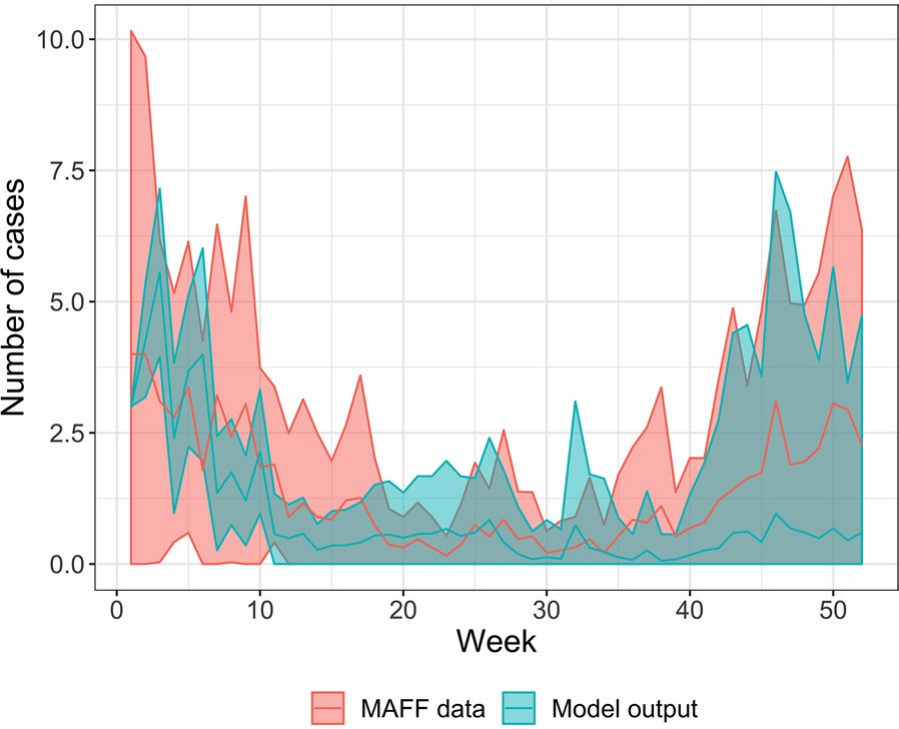
**Weekly cases of sheep scab for model particles (*n* = 100) and reported cases from 1973–1992 (*n* = 19)**. The mean (lines) and 1 standard deviation above and below the mean (shaded areas) are given for each week over a 1-year period.

Analysis shows that the reported MAFF data for the weekly outbreak incidence averaged across 1973–1992 and the averaged results from the SMC-ABC modelling were from the same statistical distribution, confirming the fit of the model (${\chi }^{2}$ = 115.3, *p* = 1) and the close visual fit between the model output and the reported MAFF data for the majority of the year is shown in Figure 2. However, this figure shows that the model output is less variable at the beginning of the year than seen in the MAFF data and the mean model result slightly underestimates the number of farm outbreaks near the end of the year, although the model output captures the broad temporal features of the data (Figure 2). The median number of weekly outbreaks of scab was 0 in both the model results (range = 0–60, IQR = 0–1, mean = 0.91, mode = 0) and in the MAFF data from 1973 to 1992 (range = 0–25, IQR = 0–2, mean = 1.42, mode = 0).

**Figure 3:**
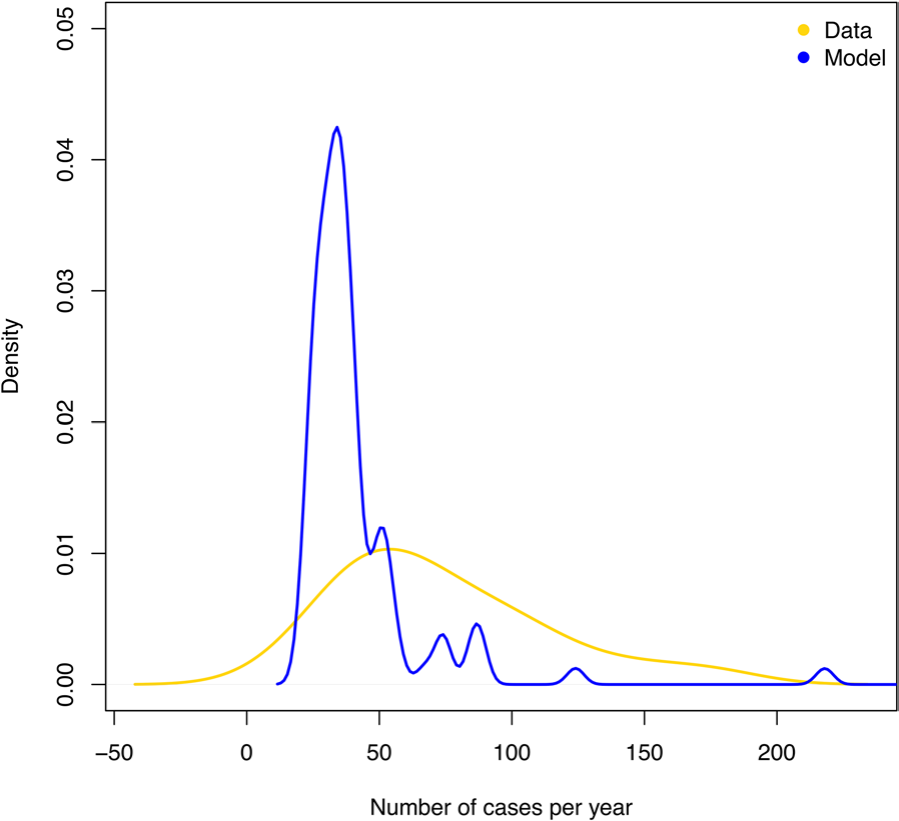
**Yearly cases of sheep scab for model particles (*n* = 100) and reported cases from 1973–1992 (*n* = 19)**. These are given as Kernel density estimates, using a gaussian smoothing kernel with the default bandwidth “nrd0”, using the density() function from the “stats” (v3.6.1) package in R.

The mean and the median number of yearly farm outbreaks of scab were lower in the model results (median = 35, mean = 47.4, mode = 34) than in the MAFF data from 1973 to 1992 (median = 67, mean = 73.7, mode = 39). The interquartile range (IQR) of the yearly outbreaks predicted by in the model result (IQR = 29.75–41.5) did not overlap with the IQR of the MAFF data from 1973 to 1992 (IQR = 43–97.5). The range of yearly outbreaks predicted by the model (21–641) was within the range of 1973–1992 MAFF data (range = 18–174). The distribution of the yearly outbreaks from the ABC model particles followed a similar normal distribution to the data, but with a long tail (Figure 3).

The estimated posterior distributions for υ, $\alpha$ and $\beta$ give more of an indication of suitable parameter values than the prior distributions with mean values per day of 2.24 (8.1^–2^ per year), 5.56 × 10^–3^ (2.1 per year) and 7.01 × 10^–2^ (25.6 per year) respectively (respective prior mean values were ~2.52 × 10^–4^, 5.62 × 10^–3^ and 6.83 × 10^–2^ per day).

**Figure 4:**
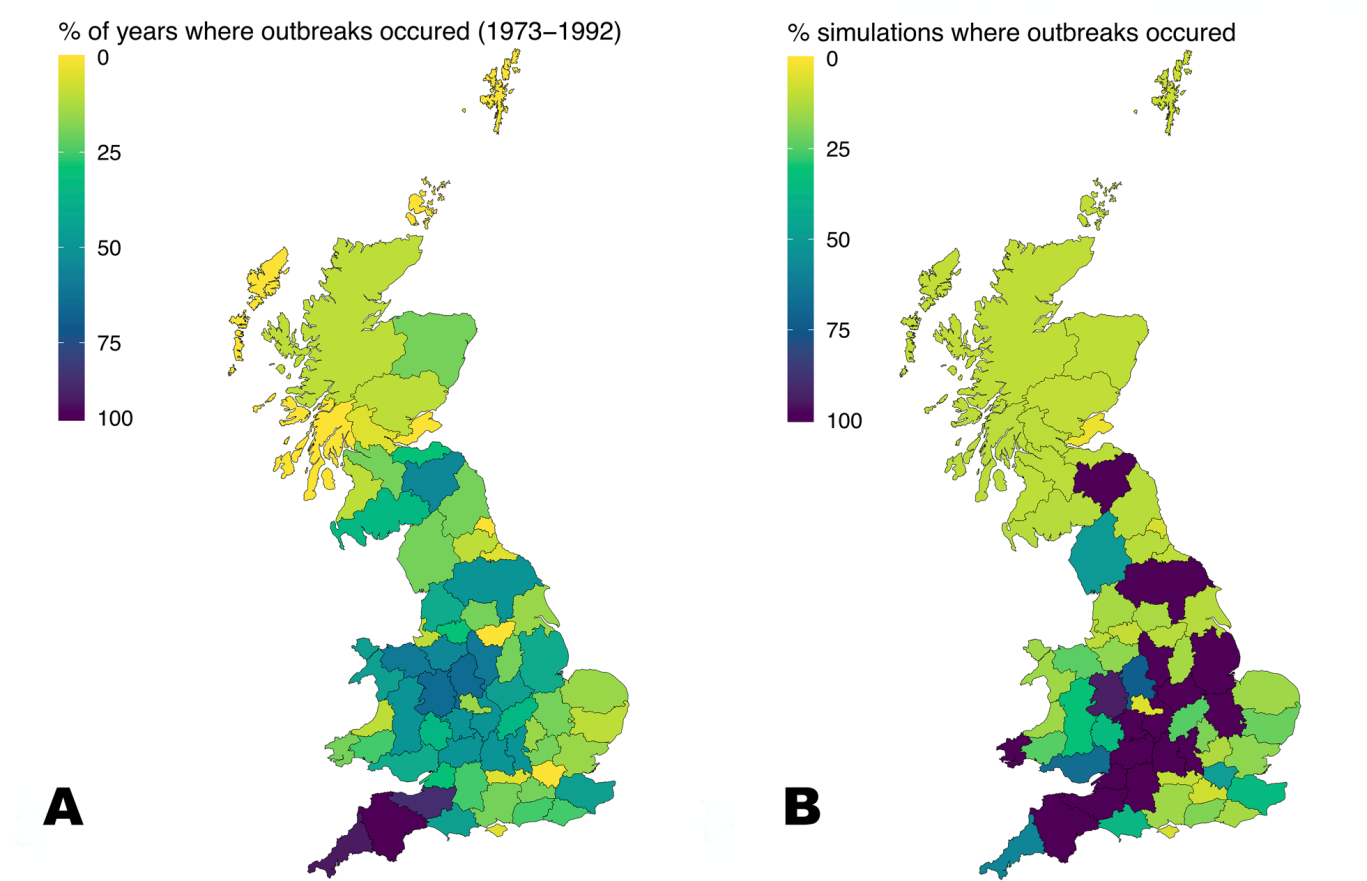
**Counties with sheep scab cases (A) in reported data (B) across repeated stochastic model simulations.** The model simulations were each run for a simulated one-year period (*n* = 3250). The reported data were sourced from the Ministry of Agriculture, Fisheries and Food (1973–1992).

The spatial location of sheep scab outbreaks (by county) predicted by the model (Figure 4) is positively correlated with the MAFF outbreak data ($\tau$ = 0.55, *p* < 0.001), with outbreaks occurring most consistently in the South West England in the 1973–1992 data (outbreaks occurred in Devon for 95% of years, 90% in Cornwall, 85% in Somerset). Outbreaks also occurred consistently in the South West of England in the model simulations (100% of simulations in Devon, 100% in Somerset), and there were 10 other counties that also had outbreaks in 100% of model simulations (see Additional file 1). For both the model simulations and the data, there were outbreaks in Scotland less consistently than in Wales and in England, although outbreaks did occur in the Scottish borders in 50% of the data years and in 100% of the model simulations.

## Discussion

A metapopulation model for sheep scab transmission within and between farms is presented here which uses authentic farm distribution data, sheep movement data and agricultural survey data for Great Britain. The model is fitted to reported data on sheep scab outbreaks between 1973 and 1992. The fitted model is shown to be able to provide a statistically significant explanation of the number of farms infected in a year as well as the observed seasonal and spatial patterns. Epidemiological models have been produced for a range of diseases in sheep, such as scrapie [8], foot and mouth [9, 10], bovine spongiform encephalopathy [11, 12] and bluetongue [13]. However, there have been no previous epidemiological models for sheep scab transmission within and between farms. One of the key features of the model developed here is that it is spatially explicit and so is able to capture the heterogeneity in the distribution and proximity of farms to each other. This is important for capturing neighbour-to-neighbour transmission between contiguous farms and the heterogeneity in long-distance movements that occur between farms that may allow for transmission across large distances. Explicit sheep movement data has previously only been analysed spatially [34] or included in network models [35, 36], but not in metapopulation models for sheep diseases. The model presented here can be used to investigate the transmission dynamics of sheep scab in any geographical region where suitable data are available.

Peak scab incidences in the northern hemisphere usually occur in the winter months [3]. Many studies have attempted to identify the factors that contribute to this highly seasonal pattern. These include a consideration of the biology of *P. ovis* mites, which survive for longer off-host in cooler and more humid conditions [37, 38], changes stress and the body condition of sheep overwinter and during gestation, and husbandry factors, such as away grazing (which may facilitate mixing) and autumn sales [3]. However, while each may be contributory there is little data on the combined impact of these. During the development of the model presented here, it was found that the incorporation of sheep movement was critical to allowing the simulations to capture the seasonality seen in the outbreak data. However, it should be noted that although the confidence intervals of the model simulations match the seasonal patterns, the mean model result underestimated the outbreak incidence seen in the data after week 40. This could be due to the fact that the outbreak data were from 1973 to 1992 whereas the movement data related to 2010. Although the general pattern of animals being moved is considered likely to be similar, some unquantified differences may exist in the transmission rate in autumn which accounts for this effect. Ideally, outbreak incidence data would have been matched with movement data for the same time-period, however unfortunately this is not possible as movement data is not available for the period 1973–1992 and scab has not been a notifiable disease in Great Britain since 1992 resulting in an absence of consistent incidence data. Even in Scotland where scab was made notifiable again in 2010 [39] it is thought that many farmers and veterinarians still do not report all outbreaks [40]. Nevertheless, the model suggests that the movement of sheep around Great Britain through markets and the movement off upland (often common grazing) to lowland winter grazing is likely to facilitate mixing of infected and naive animals and increase transmission. Clearly, managing the impact of sheep movement on scab transmission will be an important future target for optimum disease management.

Surveys to estimate the prevalence of scab in Great Britain have given cumulative flock incidences per year of 9% [4] and 8.6% [41], which were shown to vary between regions being highest in Wales, Scotland, northern and south west England. This prevalence and spatial pattern is well supported by the modelling here, although the numbers of outbreaks predicted by the model in some areas, such as Scotland, are lower than might have been anticipated from the published survey data. However, it important to note that the simulation outputs and scab incidence data represent the pattern expected following a small number of introductions in north west England, as is thought to have occurred in 1972, and the expected cumulative incidence pattern over the subsequent 20 years as scab spread. Given the pattern of sheep movements on Great Britain, an increase in incidence in Scotland may have required longer than 20 years to reach the levels seen in surveys [4, 41].

Despite multiple attempts at controlling scab by the government and the sheep industry, the disease has remained endemic in Great Britain since 1973 and is present in sheep farming systems worldwide [2]. The model presented here has the flexibility to be used to explore multiple control strategies for scab at different spatial scales and to investigate the impact of resistance to macrocyclic lactones on the prevalence of scab. In addition, it could be used to investigate the impact of novel technologies, such as a newly developed diagnostic Enzyme Linked Immunosorbent Assay [42] as part of scab interventions.

## Supplementary Information

**Additional file 1.** This includes information on how the SimInf code was adapted, how the agricultural survey data and the movements data were reconciled, detail on parameter estimation, outbreak data per county in the model simulations and in the reported data and the posterior distributions from the SMC-ABC fitting.

## Data Availability

The source code for the model is available at https://github.com/emjnixon15/ScabModel.

## Abbreviations

ABC: Approximate Bayesian Computation
AMLS: Animal Movement Licensing System
APHA: Animal and Plant Health Agency
CTMC: Continuous-time discrete Markov chains
MAFF: Ministry of Agriculture, Fisheries and Food
DEFRA: Department for the Environment, Food, and Rural Affairs
IQR: Interquartile range
OS: Ordinance Survey
SAMS: Scottish Animal Movement System
SEERAD: Scottish Executive Environment and Rural Affairs Department
SMC ABC: Sequential Monte Carlo Approximate Bayesian Computation (SMC ABC)
VLA: Veterinary Laboratories Agency
