# Supplementary material for: Sheep scab transmission: a spatially explicit dynamic metapopulation model
Source: Vet Res. 2021 Apr 12;52:54. doi: 10.1186/s13567-021-00924-y (PMC8042976; doi:10.1186/s13567-021-00924-y)
Supplement: Supplementary file 1 — Additional file 1. This includes information on how the SimInf code was adapted, how the agricultural survey data and the movements data were reconciled, detail on parameter estimation, outbreak data per county in the model simulations and in the reported data and the posterior distributions from the SMC-ABC fitting. [file 13567_2021_924_MOESM1_ESM.docx]

**Additional file 1**

**S1. Adapting SimInf**

The model was built by adapting C and R code from the existing SISe_sp model in the “SimInf” package, described fully in [1] and [2]. The original SISe_sp model is a metapopulation model of the spread of Verotoxigenic Escherichia coli O157:H7 (VTEC O157) between cattle herds in Sweden [1] and was adapted from the SISe model previously described by [3] and [4]. Here, the existing SISe_sp function was adapted to incorporate aspects of transmission specific to sheep scab. The changes to the code in the SimInf package were made and installed locally.

The SimInf package was downloaded from GitHub on the 20^th^ April 2020 and saved in a folder, the relevant C and R code adapted within this folder as outlined below and then the package installed locally by using the terminal to navigate to the folder and then using the “make install” command.

The SimInf source code is under a GNU General Public License, with permissions for commercial use, modification, distribution, patent use and private use, but with no warranty or liability included. The conditions of the permissions is that a license and copyright notice is given, that the changes made are stated, that the source is disclosed and that the modified source code is also licensed with a GNU General Public License. Therefore, the modified code presented here is also subject to the same GNU General Public License (https://www.gnu.org/licenses/gpl-3.0.en.html).

The new model code is available at [5]

**S1.1 The adapted C code for the SISe_sp model with comments and the changes made highlighted in red.**

/*

* This file is part of SimInf, a framework for stochastic

* disease spread simulations.

*

* Copyright (C) 2015 Pavol Bauer

* Copyright (C) 2017 -- 2019 Robin Eriksson

* Copyright (C) 2015 -- 2019 Stefan Engblom

* Copyright (C) 2015 -- 2020 Stefan Widgren

*

* SimInf is free software: you can redistribute it and/or modify

* it under the terms of the GNU General Public License as published by

* the Free Software Foundation, either version 3 of the License, or

* (at your option) any later version.

*

* SimInf is distributed in the hope that it will be useful,

* but WITHOUT ANY WARRANTY; without even the implied warranty of

* MERCHANTABILITY or FITNESS FOR A PARTICULAR PURPOSE. See the

* GNU General Public License for more details.

*

* You should have received a copy of the GNU General Public License

* along with this program. If not, see <https://www.gnu.org/licenses/>.

*/

#include "SimInf.h"

#include "SimInf_forward_euler_linear_decay.h"

#include "SimInf_local_spread.h"

/* Offset in integer compartment state vector */

/* Added in extra compartment here*/

enum {S, I, C};

/* Offset in real-valued continuous state vector */

enum {PHI};

/* Offsets in node local data (ldata) to parameters in the model */

enum {END_T1, END_T2, END_T3, END_T4, NEIGHBOR};

/* Offsets in global data (gdata) to parameters in the model */

/* Added new parameters called epar, tau and qprop*/

enum {UPSILON, GAMMA, ALPHA, BETA_T1, BETA_T2, BETA_T3, BETA_T4, COUPLING, EPAR, TAU, QPROP};

/**

* susceptible to infected: S -> I

*

* @param u The compartment state vector in node.

* @param v The continuous state vector in node.

* @param ldata The local data vector for the node.

* @param gdata The global data vector.

* @param t Current time.

* @return propensity.

*/

double SISe_sp_S_to_I(

const int *u,

const double *v,

const double *ldata,

const double *gdata,

double t)

{

return gdata[UPSILON] * v[PHI] * u[S];

}

/**

* infected to susceptible: I -> S (EDITED)

*

* @param u The compartment state vector in node.

* @param v The continuous state vector in node.

* @param ldata The local data vector for node.

* @param gdata The global data vector.

* @param t Current time.

* @return propensity.

*/

double SISe_sp_I_to_S(

const int *u,

const double *v,

const double *ldata,

const double *gdata,

double t)

{

return gdata[GAMMA] * (1- gdata[QPROP]) * u[I];

/* Have changed the above so that it includes infecteds only going to the susceptible - some need to become carriers */

}

/**

* infected to carriers: I -> C (NEW)

*

* @param u The compartment state vector in node.

* @param v The continuous state vector in node.

* @param ldata The local data vector for node.

* @param gdata The global data vector.

* @param t Current time.

* @return propensity.

*/

double SISe_sp_I_to_C(

const int *u,

const double *v,

const double *ldata,

const double *gdata,

double t)

{

return gdata[GAMMA] * gdata[QPROP] * u[I];

}

/**

/**

* carriers to susceptible: C -> S (NEW)

*

* @param u The compartment state vector in node.

* @param v The continuous state vector in node.

* @param ldata The local data vector for node.

* @param gdata The global data vector.

* @param t Current time.

* @return propensity.

*/

double SISe_sp_C_to_S(

const int *u,

const double *v,

const double *ldata,

const double *gdata,

double t)

{

return gdata[TAU] * u[C];

}

/**

* Update environmental infectious pressure phi

*

* Decay environmental infectious pressure phi, add contribution from

* infected individuals, carriers and proximity coupling.

* @param v_new The continuous state vector in the node after the post

* time step

* @param u The compartment state vector in the node.

* @param v The current continuous state vector in the node.

* @param ldata The local data vector for the node.

* @param gdata The global data vector.

* @param node The node.

* @param t The current time.

* @return error code (<0), or 1 if node needs to update the

* transition rates, or 0 when it doesn't need to update the

* transition rates.

*/

int SISe_sp_post_time_step(

double *v_new,

const int *u,

const double *v,

const double *ldata,

const double *gdata,

int node,

double t)

{

const int day = (int)t % 365;

const double I_i = u[I];

const double C_i = u[C];

const double N_i = u[S] + I_i + C_i; /* added in C_i here because it contributes to environmental infectious pressure. The other new compartments do not.*/

const double phi = v[PHI];

const int Nc = 3;

/* Deterimine the pointer to the continuous state vector in the

* first node. Use this to find phi at neighbours to the current

* node. */

const double *phi_0 = &v[-node];

/* Deterimine the pointer to the compartment state vector in the

* first node. Use this to find the number of individuals at

* neighbours to the current node. */

const int *u_0 = &u[-Nc*node];

/* Time dependent decay (beta) of the environmental infectious

* pressure in each of the four intervals of the year. Forward

* Euler step. */

v_new[PHI] = SimInf_forward_euler_linear_decay(

phi, day,

ldata[END_T1], ldata[END_T2], ldata[END_T3], ldata[END_T4],

gdata[BETA_T1], gdata[BETA_T2], gdata[BETA_T3], gdata[BETA_T4]);

/* Local spread among proximal nodes. */

/* Have added in the part of the local equation that includes infection from carriers */

if (N_i > 0.0) {

v_new[PHI] += (gdata[ALPHA] * I_i) + (gdata[EPAR] * gdata[ALPHA] * C_i)/ N_i +

SimInf_local_spread(&ldata[NEIGHBOR], phi_0, u_0,

N_i, phi, Nc, gdata[COUPLING]);

}

if (!R_FINITE(v_new[PHI]))

return SIMINF_ERR_V_IS_NOT_FINITE;

if (v_new[PHI] < 0.0)

return SIMINF_ERR_V_IS_NEGATIVE;

return phi != v_new[PHI]; /* 1 if needs update */

}

/**

* Run simulation with the SISe_sp model

*

* @param model The SISe_sp model.

* @param threads Number of threads.

* @param solver The numerical solver.

* @return The simulated trajectory.

*/

SEXP SISe_sp_run(SEXP model, SEXP threads, SEXP solver)

{

TRFun tr_fun[] = {&SISe_sp_S_to_I, &SISe_sp_I_to_S, &SISe_sp_I_to_C,

&SISe_sp_C_to_S};

return SimInf_run(model, threads, solver, tr_fun, &SISe_sp_post_time_step);

**S1.2 The adapted R code for the SISe_sp model with comments and changes made highlighted in red:**

## This file is part of SimInf, a framework for stochastic

## disease spread simulations.

##

## Copyright (C) 2015 Pavol Bauer

## Copyright (C) 2017 -- 2019 Robin Eriksson

## Copyright (C) 2015 -- 2019 Stefan Engblom

## Copyright (C) 2015 -- 2020 Stefan Widgren

## SimInf is free software: you can redistribute it and/or modify

## it under the terms of the GNU General Public License as published by

## the Free Software Foundation, either version 3 of the License, or

## (at your option) any later version.

##

## SimInf is distributed in the hope that it will be useful,

## but WITHOUT ANY WARRANTY; without even the implied warranty of

## MERCHANTABILITY or FITNESS FOR A PARTICULAR PURPOSE. See the

## GNU General Public License for more details.

##

## You should have received a copy of the GNU General Public License

## along with this program. If not, see <https://www.gnu.org/licenses/>.

##' Definition of the \code{SISe_sp} model

##'

##' Class to handle the \code{SISe_sp} \code{\link{SimInf_model}}.

##' @include SimInf_model.R

##' @export

setClass("SISe_sp", contains = c("SimInf_model"))

##' Create a \code{SISe_sp} model

##'

##' Create a \code{SISe_sp} model to be used by the simulation

##' framework.

##'

##' The \code{SISe_sp} model contains two compartments; number of

##' susceptible (S) and number of infectious (I). Additionally, it

##' contains an environmental compartment to model shedding of a

##' pathogen to the environment. Moreover, it also includes a spatial

##' coupling of the environmental contamination among proximal nodes

##' to capture between-node spread unrelated to moving infected

##' individuals. Consequently, the model has two state transitions,

##'

##' \deqn{S \stackrel{\upsilon \varphi S}{\longrightarrow} I}{

##' S -- upsilon phi S --> I}

##'

##' \deqn{I \stackrel{\gamma I}{\longrightarrow} S}{

##' I -- gamma I --> S}

##'

##' where the transition rate per unit of time from susceptible to

##' infected is proportional to the concentration of the environmental

##' contamination \eqn{\varphi}{phi} in each node. Moreover, the

##' transition rate from infected to susceptible is the recovery rate

##' \eqn{\gamma}, measured per individual and per unit of

##' time. Finally, the environmental infectious pressure in each node

##' is evolved by,

##'

##' \deqn{\frac{d \varphi_i(t)}{dt} = \frac{\alpha I_{i}(t)}{N_i(t)} +

##' \sum_k{\frac{\varphi_k(t) N_k(t) - \varphi_i(t) N_i(t)}{N_i(t)}

##' \cdot \frac{D}{d_{ik}}} - \beta(t) \varphi_i(t)}{

##' dphi(t)/dt=

##' alpha I / N +

##' D*sum_k(phi_k*N_k-phi_i*N_i)/(d_ik*N_i)-beta*phi_i}

##'

##' where \eqn{\alpha} is the average shedding rate of the pathogen to

##' the environment per infected individual and \eqn{N = S + I} the

##' size of the node. Next comes the spatial coupling among proximal

##' nodes, where \eqn{D} is the rate of the local spread and

##' \eqn{d_{ik}} the distance between holdings \eqn{i} and

##' \eqn{k}. The seasonal decay and removal of the pathogen is

##' captured by \eqn{\beta(t)}. The environmental infectious pressure

##' \eqn{\varphi(t)}{phi(t)} in each node is evolved each time unit by

##' the Euler forward method. The value of \eqn{\varphi(t)}{phi(t)} is

##' saved at the time-points specified in \code{tspan}.

##'

##' The argument \code{u0} must be a \code{data.frame} with one row for

##' each node with the following columns:

##' \describe{

##' \item{S}{The number of sucsceptible}

##' \item{I}{The number of infected}

##' }

##'

##' @template beta-section

##' @template u0-param

##' @template tspan-param

##' @template events-param

##' @template phi-param

##' @param upsilon Indirect transmission rate of the environmental

##' infectious pressure

##' @param epar This is the scaling rate for Carrier's contribution to transmission

##' @param tau this is the recovery rate for carriers

##' @param qprop this is the scaling rate for infecteds that become carriers

##' @param gamma The recovery rate from infected to susceptible

##' @param alpha Shed rate from infected individuals

##' @template beta-param

##' @param coupling The coupling between neighboring nodes

##' @param distance The distance matrix between neighboring nodes

##' @return \code{SISe_sp}

##' @include check_arguments.R

##' @export

##' @importFrom methods as

##' @importFrom methods is

SISe_sp <- function(u0,

tspan,

events = NULL,

phi = NULL,

upsilon = NULL,

gamma = NULL,

alpha = NULL,

epar = NULL, #new parameter epar here - this is the scaling rate for Carriers transmission

tau = NULL, #new - this is the recovery rate for Carriers

qprop = NULL, #new - this is equivalent to q. This helps scale the proportion of infecteds that recover and then become carriers

beta_t1 = NULL,

beta_t2 = NULL,

beta_t3 = NULL,

beta_t4 = NULL,

end_t1 = NULL,

end_t2 = NULL,

end_t3 = NULL,

end_t4 = NULL,

coupling = NULL,

distance = NULL) {

compartments <- c("S", "I", "C") #added in C for carriers

## Check arguments.

## Check u0 and compartments

u0 <- check_u0(u0, compartments)

## Check initial infectious pressure

if (is.null(phi))

phi <- 0

phi <- rep(phi, length.out = nrow(u0))

check_infectious_pressure_arg(nrow(u0), phi)

## Check for non-numeric parameters

check_gdata_arg(upsilon, gamma, alpha, epar, tau, qprop, beta_t1, beta_t2, beta_t3, beta_t4,

coupling) #added in new parameters

## Check interval endpoints

check_integer_arg(end_t1, end_t2, end_t3, end_t4)

end_t1 <- rep(end_t1, length.out = nrow(u0))

end_t2 <- rep(end_t2, length.out = nrow(u0))

end_t3 <- rep(end_t3, length.out = nrow(u0))

end_t4 <- rep(end_t4, length.out = nrow(u0))

check_end_t_arg(nrow(u0), end_t1, end_t2, end_t3, end_t4)

check_distance_matrix(distance)

## Arguments seem ok...go on

E <- matrix(c(1, 0, 0, 1, 1, 1), nrow = 3, ncol = 2,

dimnames = list(compartments, c("1", "2"))) #adapted this for the new model

G <- matrix(c(rep(1,16)), nrow = 4, ncol = 4,

dimnames = list(c("S -> upsilon*phi*S -> I",

"I -> gamma*((1-qprop)* I -> S",

"I -> gamma*qprop*I -> C",

"C -> tau*C -> S"),

c("1", "2", “3”, “4”)))#adapted this for the new model

S <- matrix(c(-1, 1, 0, 1, -1, 0, 0,-1,1,1,0,-1), nrow = 3, ncol = 4,

dimnames = list(compartments, c("1", "2", “3”, “4”))) #adapted this for the new model.

v0 <- matrix(as.numeric(phi), nrow = 1, byrow = TRUE,

dimnames = list("phi"))

ldata <- matrix(as.numeric(c(end_t1, end_t2, end_t3, end_t4)),

nrow = 4, byrow = TRUE,

dimnames = list(c("end_t1", "end_t2", "end_t3", "end_t4")))

ldata <- .Call(SimInf_ldata_sp, ldata, distance, 1L)

gdata <- as.numeric(c(upsilon, gamma, alpha, epar, tau, qprop, beta_t1, beta_t2,

beta_t3, beta_t4, coupling)) #added in new parameters here

names(gdata) <- c("upsilon", "gamma", "alpha", "epar", "tau", "qprop", "beta_t1", "beta_t2",

"beta_t3", "beta_t4", "coupling")

model <- SimInf_model(G = G,

S = S,

E = E

tspan = tspan,

events = events,

ldata = ldata,

gdata = gdata,

u0 = u0,

v0 = v0)

as(model, "SISe_sp")

}

#### **S2. Reconciling the agricultural survey data and the movements data**

As described in the main paper, Agricultural survey data provided by the Animal and Plant Health Agency (APHA) for England and Wales (June Census of Agriculture and Horticulture, 2010) and Scotland (June Agricultural Census, 2010) were used along with the sheep movement data to calculate the initial number of sheep at each holding at the start of the model simulation. However, since the agricultural survey takes place in June, but the movements start in January, adjustments to the numbers of sheep at each holding were made so that the agricultural survey data was reconciled with the sheep movement data. In addition, there are additional holding types, such as markets, that were not included in the agricultural survey, which have movements to and from them in the movement data.

Therefore, the number of sheep in each holding at the start of the simulation was estimated using both the agricultural survey data and the sheep movement data. This was done assuming that there were no natural births and deaths during the period of the simulation and ensuring that the number of sheep in a farm would never become negative at any point in the simulation. The two data sets had county-parish-holding (CPH) number as an identifier for holdings. The method that was used is outlined below in five steps:

- Step 1- Filtering the agricultural survey data for holdings that have at least 1 sheep. There were initially 209,881 holdings in the dataset. Of those holdings, there were 64836 that had at least 1 sheep and all of these had a unique CPH number. The only location type was “Agricultural holding”.
- Step 2- Adding CPHs which are in the movement data but not the agricultural survey data. Included all the CPH numbers that are not in the agricultural survey but are in the movement data in the survey list and allocated zero starting sheep in these holdings as it was assumed that these were markets or other locations where sheep are not kept long term. There were 112,893 holdings in the dataset at the end of this step.
- Step 3- Remove holdings where the eastings and northings were zero. After this adjustment 111,177 holdings remained in the dataset. These same holdings were also removed from the movements data which contained details of 959,335 movements prior to this adjustment and 936,204 movements following this adjustment.
- Step 4- Adjusting the starting number of sheep at holdings which have only movements away from the holdings and no movements of sheep into the holding.

1. Search across all the movements for departure CPHs which are also not destination CPHs
2. For each of these CPHs, add together the total number of sheep that move from that CPH in the year
3. Minus this number from the number of sheep in the agricultural survey.
4. If the result is negative, then add the deficit back to the survey result.

- Step 5 (see Table S1 for worked example) - Adjusting the starting number of sheep at holdings which have movements to and from the holding.

1. Calculate the cumulative measurement at time = n
2. Cumulative = cumulative at time = n-1 + movement at t = n
3. Find the minimum cumulative
4. Add this to the number of starting sheep at the CPH
5. If the result is less than zero then minus the minimum cumulative from the starting number of sheep in the agricultural survey data (as this is a negative number it will add the difference).

Following these steps there were 37 191 725 sheep in the model; before the adjustments there had been 29 236 462, which is approximately a 27% increase in sheep after the adjustment. The impact the adjustment had on the mean number of sheep per farm is shown in Table S2.

**Worked example of Step 5**

Adjusting the starting number of sheep at holdings which have movements to and from the holding***.***

#### **Table S1 A dummy example of movements for a fictional sheep holding (CPH1) to other fictional sheep holdings (CPH2-4) used in a worked example of Step 5.** Includes the first two steps of the worked example.

| Time | Movement | Step 5(i): Change in sheep population at CPH1 | Step 5(ii): Cumulative to CPH1 |
| --- | --- | --- | --- |
| 1^st^ April 2010 | CPH1 – CPH2 | -5 | -5 |
| 23^rd^ May 2010 | CPH3- CPH1 | +7 | +2 |
| 5^th^ September 2010 | CPH1 – CPH3 | -8 | -6 |
| 17^th^ December 2010 | CPH2 – CPH1 | -9 | -15 |
| 19^th^ December 2010 | CPH4- CPH1 | +20 | +5 |

Step 5(iii): The minimum cumulative from Table S1 is -15

Step 5(iv): If the survey data shows that CPH1 has 10 sheep then 10 + - 15 = -5

Step 5(v): Since the result to step iv is negative then minus thus number from the starting number 10 - - 5 = 15

If there are 15 sheep to start with in CPH1 then the number of sheep will never go negative.

**Table S2 Number of farms and mean number of sheep per farm in each country in Great Britain according to the 2010 agricultural survey.** The data provided are from after data cleaning Steps 1 to 3 were applied and after the data were reconciled to the movement data (Steps 4 and 5).

| Country | Number of Farms | Mean number of sheep per farm (After data cleaning Steps 1 to 3) | Mean number of sheep per farm (reconciled to movement data in Steps 4 and 5) |
| --- | --- | --- | --- |
| England | 68282 | 213 | 280 |
| Scotland | 22023 | 313 | 404 |
| Wales | 20727 | 376 | 443 |
| Unspecified country | 145 | 143 | 163 |
| Great Britain | 111177 | 263 | 335 |

**S3. Parameter estimation**

Table 1 in the main body of the paper shows the parameters used in the model and the justification for their values is outlined here. The majority of parameters are estimated using conditions and results from a prospective study where a sheep infected with scab was introduced into a naïve flock [6]. All parameters here are calculated as rates per day (days^-1^). In the Berriatua study, there were no deaths as a result of scab [6] and so a disease mortality rate was not included in the model.

#### S3.1 Recovery rate (γ)

The recovery rate γ can be shown (mathematically) to be:

| $\gamma= \frac{1}{average infectious period}$ |  |
| --- | --- |

assuming a constant recovery rate or an exponentially distributed infectious period.

Here, this represents the rate at which individuals recover from being highly infectious with scab (in the “I” compartment). Across the five Berritauta experiments, all transmissions occurred within the first eleven weeks (77 days) of the experiment [6]. In addition, the peak number of mites on index cases were seen on week 11 on average, when plotting the number of mites on index cases over time, from when lesions were first established on the index case (prior to the experiment) to the end of the experiment (these data were only available for index cases 2A, 2B and 2C). Therefore, 11 weeks is assumed to be the average period of infection for the acute phase. After this point, the index cases of infected sheep either recovered from scab and became susceptible again or had a lower population size of *P.ovis* mites and were less infectious and considered to be “Carriers” (Figure 1 from [6]). Therefore, the rate at which individuals recover from being highly infected in the model is assumed to be:

$\gamma= \frac{1}{77}$ days^-1^

#### S3.2 The proportion of acute infections that become carriers (q)

The acute infectious cases that recover at rate $\gamma$ either become carriers of scab, or susceptible. In the model, $\gamma$ is scaled by the proportion of acute infections that become carriers (*q*).

At the end of the Berriatua study [6], two out of four index cases still had mites at the end of the study (the index case from Trial 1 is not included since it was removed four weeks into the study). This is used to calculate the proportion of acute infections (in disease state “I”) that become carriers of scab (disease state “C”). Therefore:

$$q= \frac{2}{4}$$

Those that recover without becoming carriers *(1-q)* recover from scab and become instantly susceptible. This is reflective of the other two out of four index cases which had no mites at the end of the study.

#### S3.3 Recovery rate for carriers ($\tau)$

It has been suggested that the period of infection (including both the acute infectious stage and the carrier stage) for sheep scab without treatment may be at least two years [7]. Although clinical signs might not be present on an infected individual for the whole two-year period, mites can remain concealed within cryptic sites on a sheep, such as hidden skin folds or the ear [7, 8] and even with only one pregnant female mite present, if passed to another host, will establish an infection on that host [9].

As discussed in section S3.1, the period of infection for the acute phase is thought to be approximately eleven weeks. Therefore, it is assumed that the period of infection for the carrier phase is the total period of infection (two years and assuming neither are leap years), minus eleven weeks. Therefore, the rate at which carriers recover and become susceptible is assumed to be:

$\tau=\frac{1}{653}$days^-1^

#### S3.4 Reduced transmission of scab by carriers $(\varepsilon)$

When individuals are considered to be carriers of scab, the mite population they harbour is past its peak size and therefore carriers are less likely to transmit mites to other individuals (directly or via the environment) compared to acute infected sheep with higher numbers of mites. This is captured in the model by the use of a scaling factor $\left( \varepsilon\right)$for the transmission rate (β).

In the Berriatua study [6], all transmission occurred during the first eleven weeks of the experiments, which in the model, is considered to be when individuals are “Infected” and includes the period where the mite population reaches its peak. For the two index cases (2B and 2C) with mites at the end of the experiment, the number of mites they have at week 12 is about one third compared to the average number of mites they had in the previous eleven weeks (Figure 2 from [6]).

Therefore, it is assumed here that the rate of transmission for carriers is approximately one third of the transmission rate for acute infected individuals:

$$\varepsilon= \frac{1}{3}$$

#### S3.5 The Euclidean distance between holding i and neighbour k (d_ik_)

A maximum threshold for *d_ik_* was set to 2 km. The holding location coordinates indicate the centre of a holding, so assuming all holdings are an equal circular area, the radius of the farm itself (~517m, area = 0.86 km2 [10]), plus the radius of an adjacent farm is 1.4 km in total. This is rounded up to 2 km as farms are not actually circular in shape and because a CPH can cover land and buildings up to 16 km away from the main livestock handling area [11].

#### S3.6 Estimating the prior distributions for $\alpha$, $\beta$ and 𝜐

For each parameter ($\alpha$, $\beta$ and *𝜐*), a one-at-a-time sensitivity analysis (OAT SA) was performed where the model was run repeatedly for different values of the parameter of interest under the conditions described in the main body of text. For each OAT SA, the model was run 5 times with different seeds. The yearly incidence from the model outputs were then compared to the yearly incidence from 1976 in the MAFF data (101 new outbreaks) using the Poisson Log Likelihood:

| data*log(model) - model | (Equation S1) |
| --- | --- |

The values of the unknown parameters ($\alpha$, $\beta$ and *𝜐*) when they were not the parameter of interest were estimated prior to performing the OAT SA by running the model until the estimates for these parameters gave yearly incidence results which were the same order of magnitude as the yearly incidence from 1976 in the MAFF data (101 new outbreaks). The best estimates were 0.004 for $\alpha$, 0.07 for $\beta$ and 0.012 for upsilon. All other parameters used were as described in Table 1 in the manuscript.

In the OAT SA for $\alpha$ (the daily contribution to environmental pressure per infected individual), $\alpha$ was varied from 0.001 to 0.02. by 0.001. The Poisson log likelihood suggests that the model output deviates the least from the data when $\alpha$ is within the range of 0 to 0.012 (Figure S1). Therefore, in the ABC SMC, the prior distribution for $\alpha$ is a uniform distribution with a minimum value of 0 and a maximum value of 0.012 (Table 1).

In the OAT SA for $\beta$ (the decay rate of the environmental infectious pressure), ($\beta$) was varied from 0 to 1 by 0.01 in an OAT SA and from 0 to 0.2 by 0.01. The Poisson log likelihood suggests that the model output deviates the least from the data when $\beta$ is within the range of 0.07 to 0.1 (Figure S2, Figure S3). This distribution was reduced by looking at the death rate of *P.ovis* mites. Since the environmental infectious pressure captures direct and indirect transmission, it was assumed that individual *P.ovis* mites may survive on a host for a maximum of 40 days [12] or remain viable in the environment for 15 days [13]. Therefore, it was assumed the decay rate for mites would not be much lower than 0.025 (1/40) or much greater than 0.07 (1/14). The prior distribution was therefore estimated to be uniform with a minimum value of 0.02 and a maximum value of 0.08.

In the OAT SA for *𝜐* (the indirect transmission rate from the environmental compartment *j* to susceptible sheep in holding *i*), *𝜐* was varied from 0 to 0.001 by 0.0001 in an OAT SA. The Poisson log likelihood suggests that the model output deviates the least from the data when $\upsilon$is within the range of 0 to 0.0006 (Figure S4). Therefore, in the ABC SMC, the prior distribution for α is a uniform distribution with a minimum value of 0 and a maximum value of 0.0006.


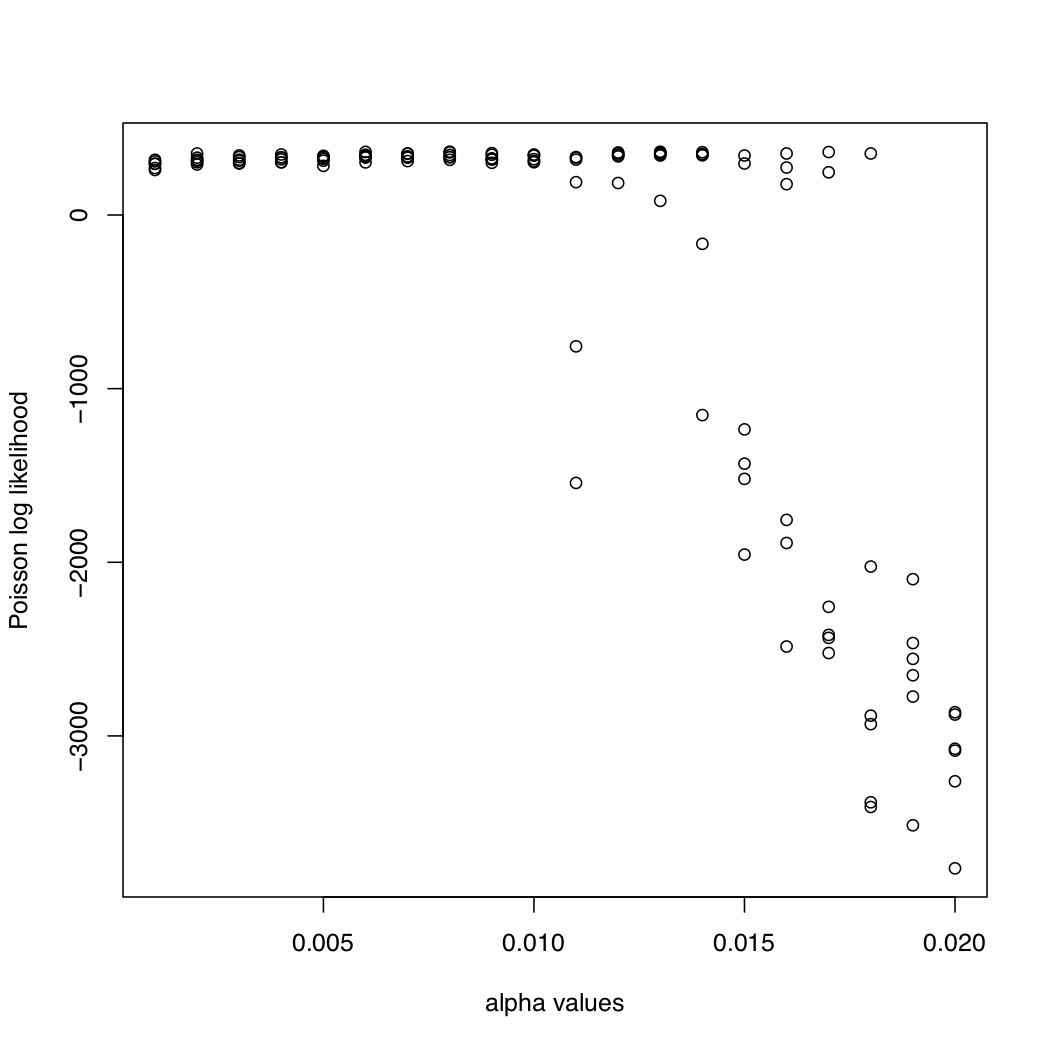


$\alpha$ value

**Figure S1.** **Poisson log likelihood of yearly incidence between the model output and the MAFF data from 1976 when the daily contribution to environmental pressure per infected individual** ($\alpha)$ **is varied from 0 to 0.02 by 0.001 in one-at-a-time sensitivity analysis.**

*
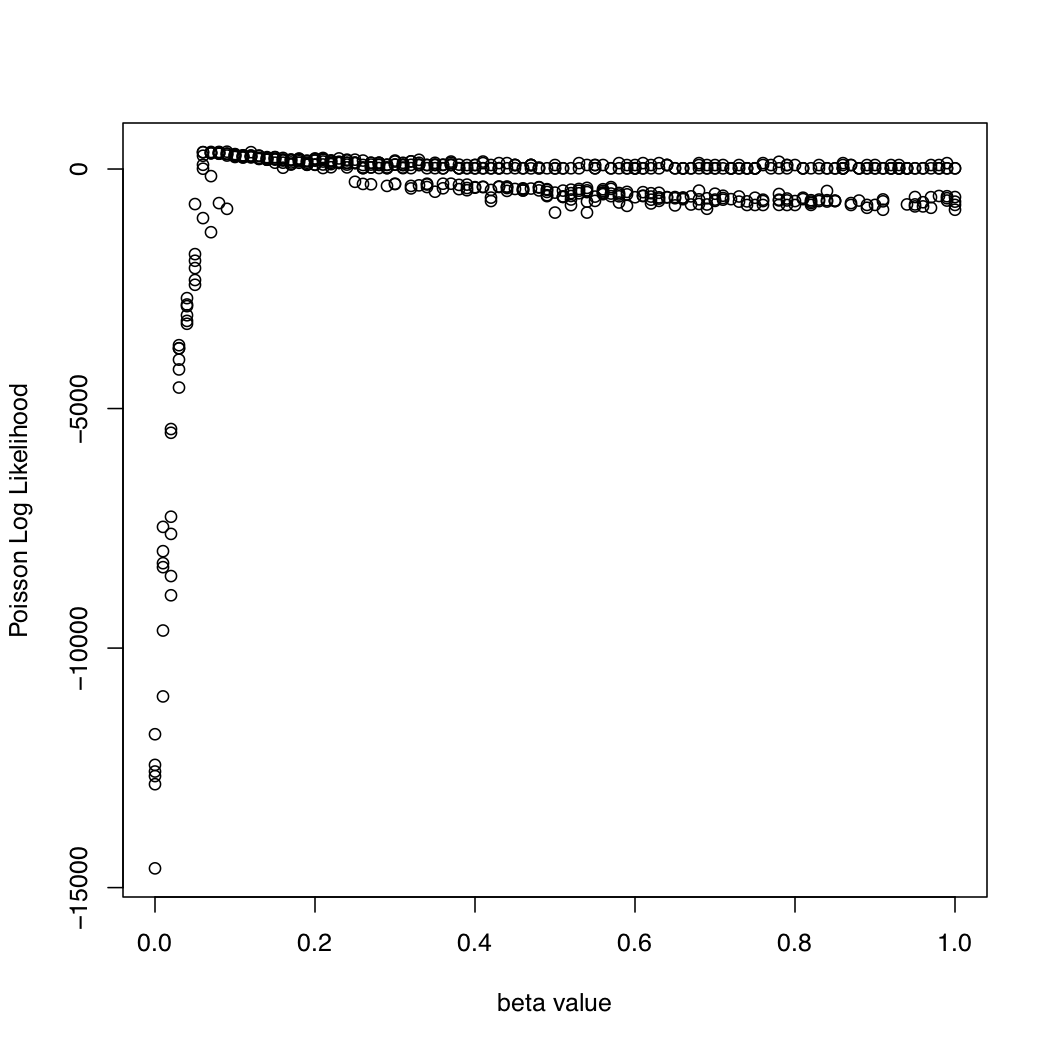
*

$\beta$ value

**Figure S2 Poisson log likelihood of yearly incidence between the model output and the MAFF data from 1976 when the decay rate of the environmental infectious pressure (**$\boldsymbol{\beta)}$ **is varied from 0 to 1 by 0.001 in one-at-a-time sensitivity analysis.**

*
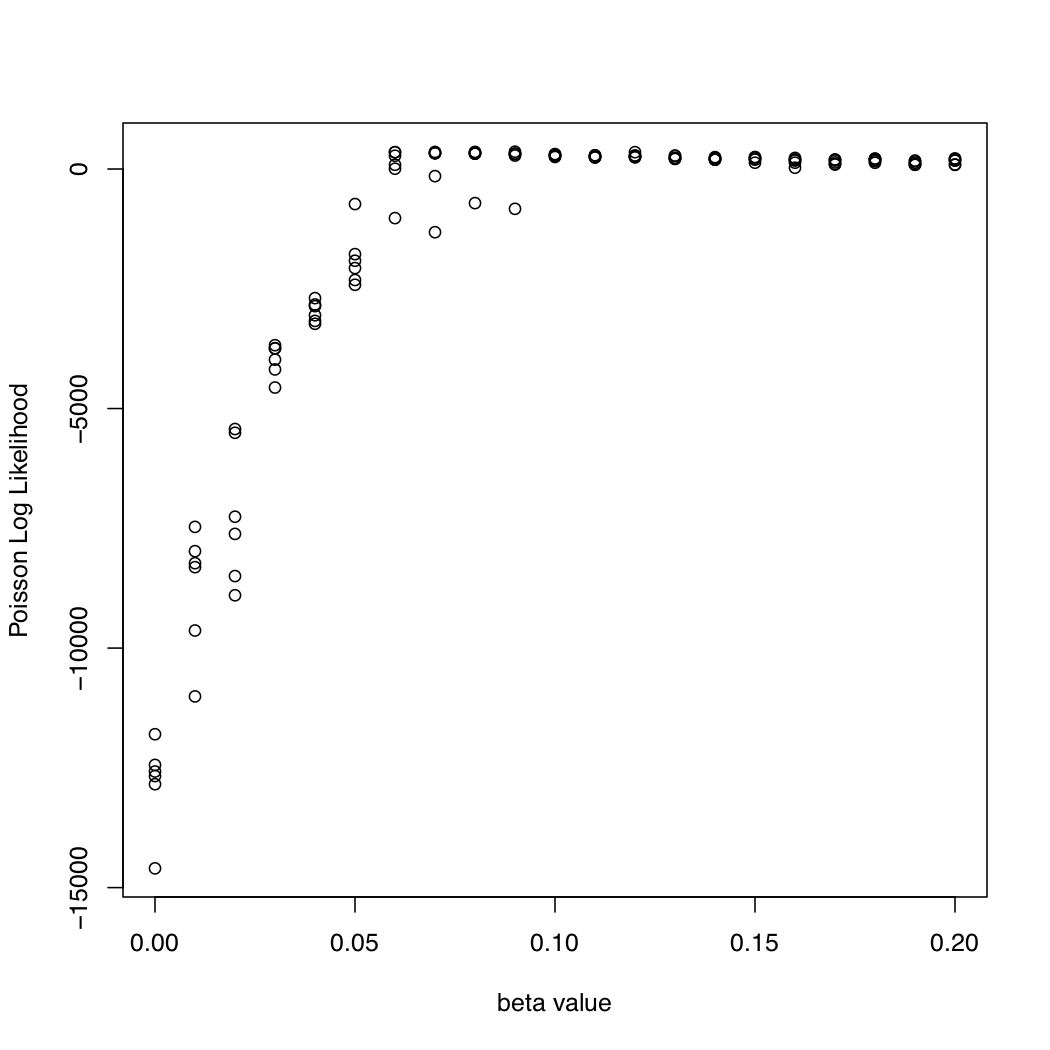
*

$\beta$ value

**Figure S3. Poisson log likelihood of yearly incidence between the model output and the MAFF data from 1976 when the decay rate of the environmental infectious pressure (**$\beta)$ **is varied from 0 to 0.2 by 0.001 in one-at-a-time sensitivity analysis.**

***
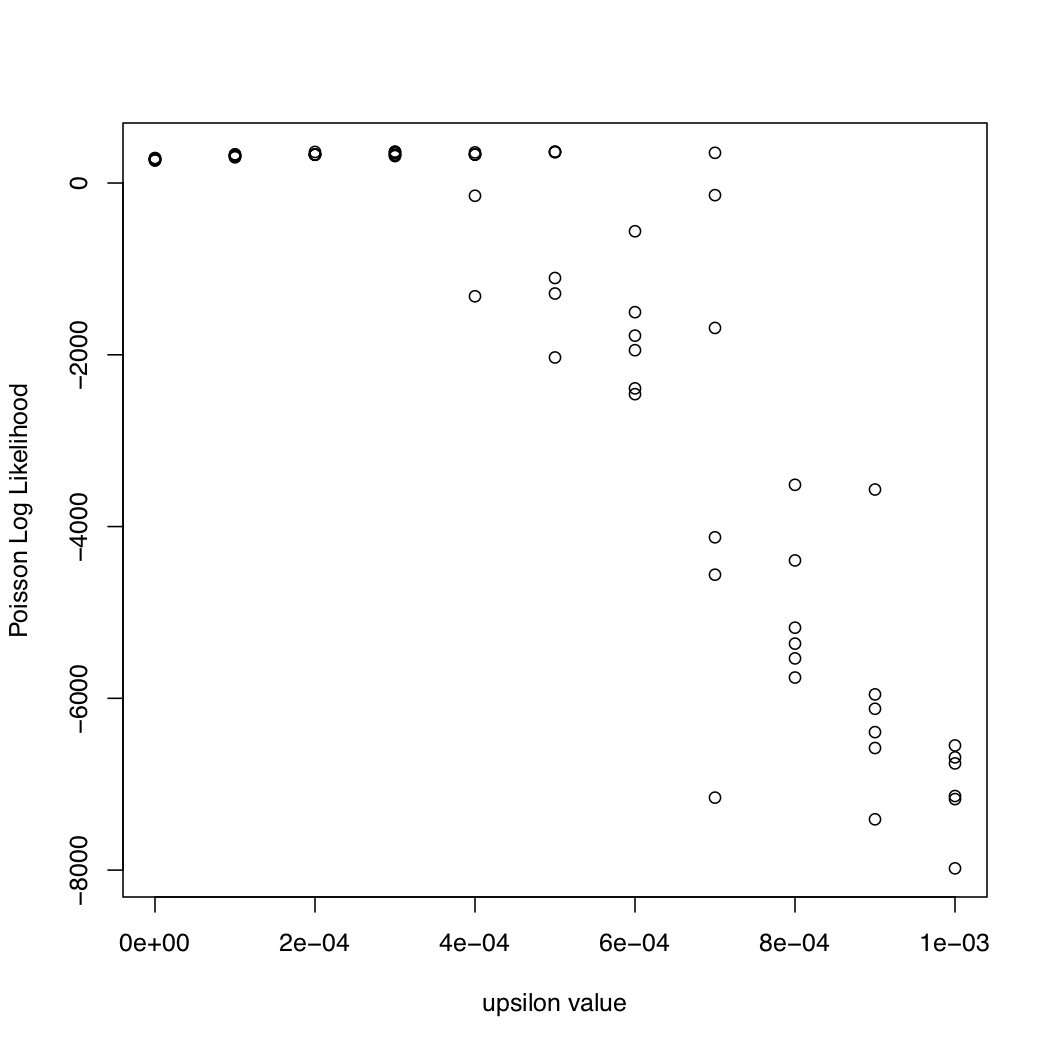
***

$$\upsilon$$

**Figure S4. Poisson log likelihood of yearly incidence between the model output and the MAFF data from 1976 when the indirect transmission rate from the environmental compartment j to susceptible sheep in holding i** *𝜐* **is varied from 0 to 0.001 by 0.0001 in one-at-a-time sensitivity analysis.**

#### S3.7 Estimating the spatial coupling

The SimInf model has been written so the contribution to infectious pressure from a neighbouring farm *k* to farm *i* is scaled by a transmission multiplier that is based on the spatial coupling parameter (*D*) divided by the distance between the two holdings (*d_ik_)* (Equation 1, main body of text). The nature of this means that between-farm transmission via spatial proximity can never be greater than within-farm transmission.

The spatial coupling parameter (*D)* was modified in a one-at-a-time sensitivity analysis (from 0, to 1, by 0.05) while keeping all unknown parameters as described in section S3.6 ($\alpha$ = 0.0004, *𝜐* = 0.012 and $\beta$ = 0.07) and all other parameters as shown in Table 1 in the manuscript (Figure S5). A poisson log likelihood (equation S1) comparing the yearly incidence results from the OAT SA for spatial coupling and the yearly incidence from 1976 in the MAFF data (101 new outbreaks) was carried out and the top 5% results presented (Figure S5B). However, it was unclear from these results which range of the parameter value would be most suitable when using a poisson log likelihood. This result is similar to the result seen in a sensitivity analysis of the spatial coupling parameter in the original SISe_sp model by [1], where they also found that halving or doubling the value of *D* did not have a large impact on the model fit, however, removing it from the model did impact the model fit.

Therefore, it was decided here to use the mean of the top of the poisson log likelihood results as the spatial coupling parameter (*D*) (0.5642857).


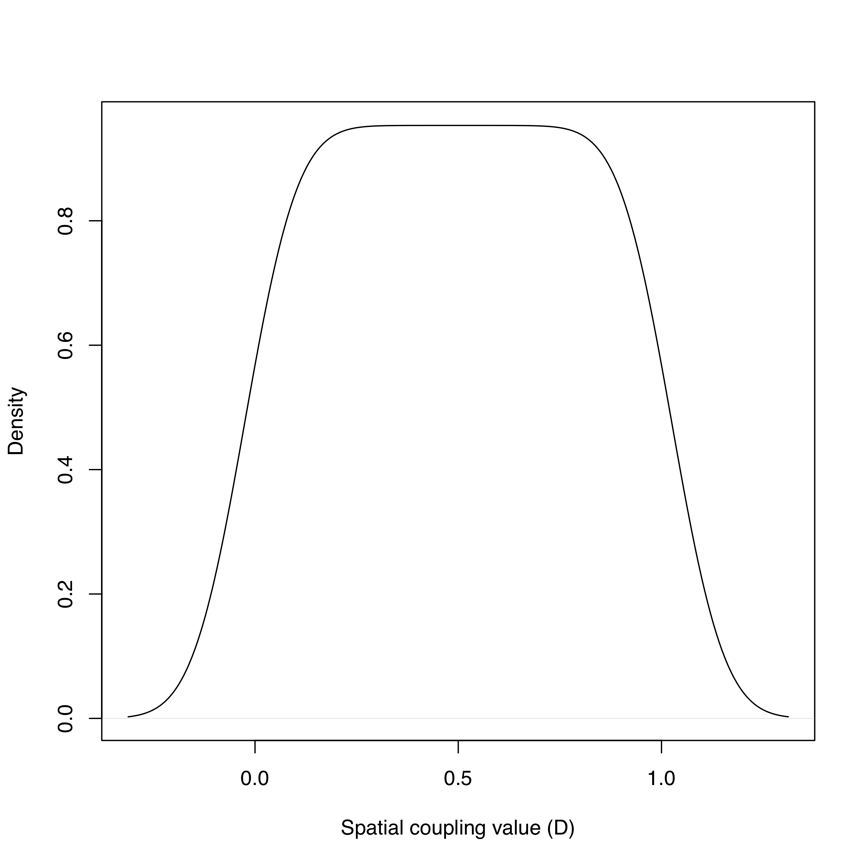

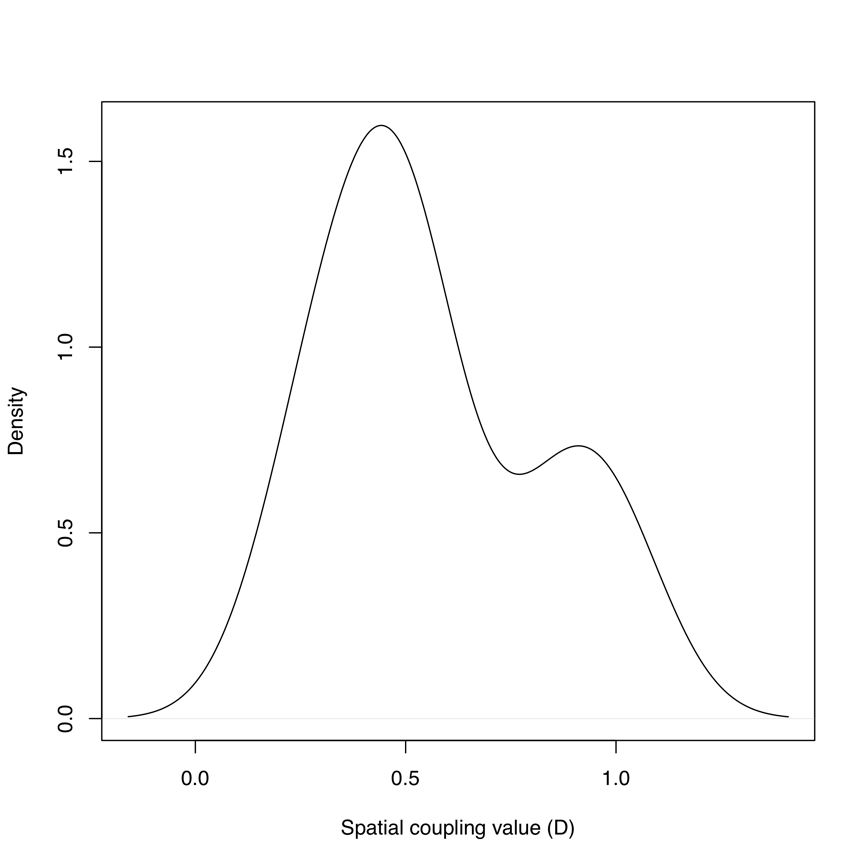


**(B)**

**(A)**

**Figure S5 Density of model output when the spatial coupling (D) is varied from 0 to 1 by 0.05 in one-at-a-time sensitivity analysis. (A)** All results **(B)** Top 5% of results from a poisson log likelihood of yearly incidence between the model output and the MAFF data from 1976.

**S4. Outbreak data per county in the model simulations and in the reported data from 1973-1992.**

The percentage of years (in the reported data from the Ministry of Agriculture, Fisheries and Food) or simulations (from the model) where an outbreak of sheep scab occurred in each county in Great Britain is given in Table S3.

**Table S3. The percentage of years (in the reported data from the Ministry of Agriculture, Fisheries and Food) or simulations (from the model) where an outbreak of sheep scab occurred in each county in Great Britain.**

| **County** | **Percentage of years where there was an outbreak (1973-1992)** | **Percentage of model simulations where there was an outbreak (n=3250)** |
| --- | --- | --- |
| Argyll & Bute | 0 | 11 |
| Ayrshire | 10 | 11 |
| Bedfordshire | 15 | 20 |
| Berkshire | 5 | 10 |
| Buckinghamshire | 50 | 100 |
| Cambridgeshire | 20 | 100 |
| Carmarthenshire | 25 | 24 |
| Ceredigion | 10 | 15 |
| Cheshire | 55 | 17 |
| Cleveland & Darlington | 5 | 11 |
| Clyde Valley | 20 | 11 |
| Cornwall and Isles of Scilly | 90 | 59 |
| Cumbria | 20 | 50 |
| Derbyshire | 60 | 100 |
| Devon | 95 | 100 |
| Dorset | 45 | 37 |
| Dumfries & Galloway | 35 | 12 |
| Durham | 10 | 11 |
| East Central | 5 | 11 |
| East Riding & North Lincolnshire | 15 | 12 |
| East Sussex | 25 | 14 |
| Eileanan an lar | 0 | 10 |
| Essex | 15 | 17 |
| Fife | 0 | 3 |
| Gloucestershire | 50 | 100 |
| Greater London | 0 | 49 |
| Greater Manchester | 30 | 10 |
| Hampshire | 20 | 15 |
| Herefordshire | 35 | 35 |
| Hertfordshire | 20 | 13 |
| Highlands | 10 | 10 |
| Isle of Wight | 5 | 7 |
| Kent | 45 | 35 |
| Lancashire | 40 | 14 |
| Leicestershire and Rutland | 45 | 100 |
| Lincolnshire | 40 | 100 |
| Lothian | 30 | 11 |
| Merseyside | 10 | 11 |
| Norfolk | 15 | 15 |
| North East Wales | 60 | 24 |
| North Eastern Scotland | 20 | 10 |
| North West Wales | 45 | 15 |
| North Yorkshire | 50 | 100 |
| Northamptonshire | 35 | 25 |
| Northern Somerset & South Gloucestershire | 30 | 100 |
| Northumberland | 20 | 11 |
| Nottinghamshire | 20 | 14 |
| Orkney | 0 | 10 |
| Oxfordshire | 50 | 100 |
| Pembrokeshire | 20 | 100 |
| Powys | 50 | 31 |
| Scottish Borders | 55 | 100 |
| Shetland | 0 | 8 |
| Shropshire | 65 | 94 |
| Somerset excl North | 85 | 100 |
| South Wales | 40 | 67 |
| South Yorkshire | 0 | 12 |
| Staffordshire | 65 | 73 |
| Suffolk | 10 | 22 |
| Surrey | 20 | 8 |
| Tayside | 10 | 10 |
| Tyne & Wear | 0 | 6 |
| Warwickshire | 50 | 100 |
| West Midlands | 15 | 6 |
| West Sussex | 25 | 19 |
| West Yorkshire | 20 | 14 |
| Wiltshire | 25 | 100 |
| Worcestershire | 50 | 100 |

**S5. The posterior distributions from the SMC-ABC fitting**

The prior and estimated posterior distribution given by the SMC-ABC algorithm for parameters used in model fitting, $\upsilon$, the indirect transmission rate from the environmental compartment to susceptible sheep, $\alpha$, the daily contribution to environmental pressure per infected individual) and $\beta$, the decay rate of the environmental infectious pressure given by the SMC-ABC algorithm are given in Figure S6.

**(A)**

**(C)**

**(B)**

**Figure S6 The estimated posterior distribution given by the SMC-ABC algorithm for parameters (A)** $\upsilon$, the indirect transmission rate from the environmental compartment to susceptible sheep (Prior(0,0.0006, uniform))**(B)** $\alpha$, the daily contribution to environmental pressure per infected individual (Prior(0,0.012,uniform)) and **(C)** $\beta$, the decay rate of the environmental infectious pressure given by the SMC-ABC algorithm ((Prior (0.02, 0.08, uniform)).

**Bibliography**

1. Widgren S, Engblom S, Emanuelson U, Lindberg A (2018) Spatio-temporal modelling of verotoxigenic *Escherichia coli* O157 in cattle in Sweden: exploring options for control. Vet Res 49:78
2. Widgren S, Bauer P, Eriksson R, Engblom S (2019) SimInf: An R package for data-driven stochastic disease spread simulations. J Stat Softw 91:1-42
3. Bauer P, Engblom S., Widgren S (2016) Fast event-based epidemiological simulations on national scales. The Int J High Perform Comput Appl 30:438-453
4. Widgren S, Engblom S, Bauer P, Frossling J, Emanuelson U, Lindberg A (2016) Data-driven network modelling of disease transmission using complete population movement data: spread of VTEC O157 in Swedish cattle. Vet Res 47: 81
5. Nixon EJ (2021) ScabModel. GitHub repository. <https://github.com/emjnixon15/ScabModel> Accessed 17 March 2021
6. Berriatua E, French NP, Wall R, Smith KE, Morgan KL (1999) Within-flock transmission of sheep scab in naive sheep housed with single infested sheep. Vet Parasitol 83:277-289
7. Babcock O G, Black WL (1933) The common sheep scab mite and its control. Texas Agricultural Experiment Station Bulletin 479:1-34
8. Bates P G (1997) The Pathogenesis and ageing of sheep scab lesions- Part 1. State Veterinary Journal 7:11-15
9. van den Broek AH, Else RW, Huntley JF, Machell J, Taylor MA, Miller HRP (2004) Early innate and longer-term adaptive cutaneous immune-inflammatory responses during primary infestation with the sheep scab mite, *Psoroptes ovis*. JComp Pathol 131(4):318-329
10. Eurostat statistics explained (2013) Agricultural census in the United Kingdom <http://ec.europa.eu/eurostat/statistics-explained/index.php/File:Table_Farm_Structure_key_indicators_UK_2000_2010.PNG> Accessed 18 December 2020
11. Department for Environment, Food and Rural Affairs, 2018. Register land you use to keep livestock <https://www.gov.uk/guidance/register-land-you-use-to-keep-livestock> Accessed 18 December 2020
12. Wall R, Smith KE, Berriatua E, French NP (1999) Simulation analysis of the population dynamics of the mite, *Psoroptes ovis*, infesting sheep. Vet Parasitol 83:253-264
13. O'Brien DJ, Gray JS, O'Reilly PF (1994) Survival and retention of infectivity of the mite Psoroptes ovis off the host. Vet Res Commun 18:27-36
